# Phenotypic Expression of *Salmonella enterica* Due to Environmental Stress

**DOI:** 10.3390/microorganisms14040748

**Published:** 2026-03-26

**Authors:** Prantho Malakar Dipta, Seth Adesope, Eniola Betiku, Tomi Obe

**Affiliations:** 1 Department of Poultry Science, University of Arkansas System Division of Agriculture, Fayetteville, AR 72701, USA; pdipta@uark.edu (P.M.D.); sadesope@uark.edu (S.A.); ebetiku@uark.edu (E.B.); 2 Department of Food Science, Center for Food Safety, University of Arkansas System Division of Agriculture, Fayetteville, AR 72704, USA

**Keywords:** *Salmonella enterica*, stress-conditioning, PAA, QAC, biofilm, antibiotic resistance

## Abstract

*Salmonella enterica* remains a major food safety concern in poultry, and processing-related stress can influence its survival and persistence. This study evaluated the phenotypic expression of *S. enterica* serotypes Kentucky (SK), Infantis (SI), Schwarzengrund (SS), and Typhimurium (ST) following antimicrobial and temperature stressors. A pre-harvest isolate of each serotype was gradually exposed to increasing concentrations of peracetic acid (PAA) and quaternary ammonium compounds (QACs), starting at 40 ppm and 1 ppm, respectively, until minimum inhibitory and bactericidal concentrations (MICs/MBCs) were established. Stressed cells were then subjected to cold (4 °C, 60 min) and heat (55 °C, 6 min) shock and assessed for sanitizer tolerance, biofilm formation and recovery, and antibiotic resistance. Sanitizer tolerance after daily conditioning varied among *S. enterica* serotypes, with ST and SK showing the highest tolerance to PAA and QACs, respectively. The tolerance of PAA variants increased by 10–20 ppm and QAC variants by 2–8 ppm following stress exposure. The double-stressed variants of ST significantly (*p* < 0.05) formed more biofilm than the control after PAA, whereas no significant differences were observed among the variants for other serotypes. Biofilm recovery was higher for the stressed variants of SI and SS (*p* < 0.05) following PAA stress but remained the same across all serotypes after QAC stress. QAC-stressed variants showed more phenotypic changes across the antibiotics tested. Notably, the stressed variants of SK, SS, and ST displayed increased MICs, including a 2- to 4-fold rise in azithromycin for the SK and ST variants. There was an increase in the MICs of ceftriaxone and nalidixic acid for some SK and SS variants. These findings suggest that environmental stress can significantly enhance the tolerance, survival, and persistence of *S. enterica*.

## 1. Introduction

*Salmonella enterica* is a leading cause of bacterial foodborne illness and a public health threat, with an estimated 1.35 million cases of salmonellosis each year in the United States [1,2]. The European Union One Health 2024 zoonoses data also reported 18.6 cases of salmonellosis per 100,000 population, underscoring the global public health significance of this foodborne pathogen [3]. *Salmonella* serotypes vary in their natural reservoirs and host ranges, as well as geographic and seasonal distributions, and ability to cause infection [4,5,6,7]. The Centers for Disease Control and Prevention (CDC) has identified more than 1300 serotypes linked to human illness in the U.S., and in contrast, more than 2500 serotypes have been documented worldwide [6].

Studies have shown an increasing trend in *Salmonella* outbreaks and infections linked to contaminated poultry and poultry products, which remain major reservoirs for *Salmonella* and leading sources of salmonellosis [8,9]. Control measures for salmonellosis have proved difficult owing to the emergence of virulent serotypes from diverse sources and multiple transmission routes [10,11]. Numerous studies analyzing surveillance data from the U.S. Department of Agriculture’s (USDA) Food Safety Inspection and Services (FSIS) consistently report that the top five most detected non-typhoidal *Salmonella enterica* serotypes in U.S. poultry establishments are Kentucky, Enteritidis, Infantis, Schwarzengrund, and Typhimurium [12,13,14]. Of these serotypes, Enteritidis, Infantis, Schwarzengrund, and Typhimurium are among the most frequently reported in foodborne outbreaks. Over the last decade, these serotypes, have remained prevalent in U.S. poultry production [13,14], while Enteritidis and Typhimurium have continued to be associated with multistate outbreaks of salmonellosis [15,16]. According to the FSIS quarterly data, the prevalence of serotype Kentucky from domestic chicken across the U.S. reduced from 30% in 2020 (FY 2021, Q1) to 19% in 2025 (FY 2025, Q2); however, it remained the top serotype commonly isolated from poultry [17]. While *S*. Kentucky is not a human pathogen, the reported high prevalence of multidrug-resistant strains suggests a potential threat to public health [18,19]. The most notable serotypes in the surveillance data are Infantis and Schwarzengrund that display distinct patterns. *S*. Infantis showed a striking upward rise in prevalence, increasing from 4.6% in FY 2016 (Q1) to 33.6% in FY 2025 (Q2). In contrast, *S*. Schwarzengrund appeared sporadically over the same period [17]. Moreover, *S*. Infantis was previously considered an emerging serotype with a high degree of tolerance to antimicrobials [20,21] but has been confirmed as a virulent serotype increasingly linked to human infection globally and often demonstrating multi-drug resistance [21,22,23].

Antimicrobial resistance (AMR) in non-typhoidal *Salmonella* is a major public health threat due to reduced susceptibility to medically important antibiotics, especially those classified as highest priority by World Health Organization [24]. This includes reduced susceptibility to fluoroquinolones and resistance to third-generation cephalosporins. The carriage of pESI megaplasmid has been reported to confer high levels of AMR in serotype Infantis, as the plasmid confers resistance to multiple antibiotic classes, including extended-spectrum β-lactamase (ESBL) [21,25]. In addition, this unique plasmid could contribute to increased biofilm formation and adhesion in strains co-harboring fimbriae and adhesion genes, thereby enhancing persistence and pathogenicity [26,27]. Genetic determinants of AMR are critical for the persistence and pathogenicity of any serotype; however, persistence can also occur through non-genetic adaptations, such as exposure to environmental stress [28,29]. Stress response to different environmental exposure significantly affects bacterial survival and persistence, posing new challenges for microbial management [30]. It is crucial to evaluate how pre-exposure to environmental stressors, including sanitizers, disinfectants, and temperature extremes, affect *Salmonella*’s resilience. Aside from *S*. Infantis, *S*. Schwarzengrund has also emerged globally as a virulent contender with a strong association to resistance against medically important antibiotics such as β-lactams and fluoroquinolones [31].

In poultry processing environments, peracetic acid (PAA) and quaternary ammonium compounds (QACs) are widely used as disinfectants and sanitizers to reduce microbial load and enhance food safety [32,33]. However, the overuse or improper application of these chemicals could influence bacterial tolerance and survival, leading to the expression of adaptive mechanisms and new challenges for food safety [34,35]. These adaptive mechanisms allow *Salmonella* to withstand sanitizer pressure, shifting its phenotypic characteristics and virulence [36]. PAA is a recognized oxidizer widely used during chilling operations, and QACs are often used for sanitizing operations [33]. However, improper application and exposure to sub-inhibitory concentrations can cause injury, stimulating an adaptive response that increases tolerance and virulence [37]. This increase in virulence was documented by Fruci and Poole [38], as an increase in the ability of the exposed cells to (i) withstand higher concentrations of similar (homologous adaptation) and different (heterologous adaptation) compounds [39], (ii) display changes in cell morphology, termed stress-induced morphogenesis [40], (iii) changes in growth dynamics [41], and (iv) cross-protect against other antimicrobials or antibiotics [42,43,44]. Moreover, exposure to one stressor could lead to cross-tolerance to other environmental stressors, further influencing survival and persistence in processing environments [45].

The stress-adaptation mechanism underlying increased tolerance of *Salmonella enterica*, particularly in the context of increased tolerance to antibiotic, remains poorly understood. This gap necessitates investigation into phenotypic and genotypic traits of stress-exposed *Salmonella*. Given this background information, we hypothesized that environmental stress could enhance *Salmonella* serotypes for tolerance and survival. Therefore, the aim of our study was to define phenotypic changes in *Salmonella enterica* serotypes caused by environmental stress commonly found in poultry processing environments and highlight differences between stressed and unstressed variants within and between the serotypes.

## 2. Materials and Methods

### 2.1. Experimental Design

**Figure 1:**
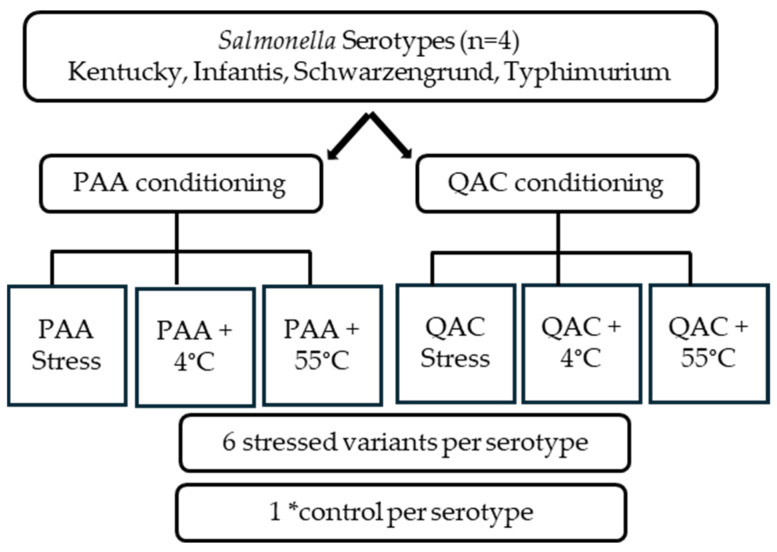
Schematic of experimental design. Antimicrobials used were peracetic acid (PAA) and quaternary ammonium compounds (QACs). The *control is the unexposed strain.

Isolates of four *Salmonella* serotypes linked to poultry (Kentucky, Infantis, Schwarzengrund, and Typhimurium) were used in this study and each serotype was represented by one well-characterized isolate. The isolates were exposed to sublethal concentrations of two antimicrobials commonly used during poultry processing, namely peracetic acid (PAA, 24%) and quaternary ammonium compounds (QACs, 20%). Both sanitizers were obtained locally and manufactured by Fortrex, Inc. Ltd. (North Little Rock, AR, USA). The resulting variants (PAA-stressed or QAC-stressed) were further subjected to cold stress at 4 °C for 60 min and heat stress at 55 °C for 6 min, reflecting the scalding and chilling steps of poultry processing (Table 1). This produced six stressed variants per serotype, namely PAA-stressed (P), PAA-stressed with 4 °C (P4), PAA-stressed with 55 °C (P55), QAC-stressed (Q), QAC-stressed with 4 °C (Q4), and QAC-stressed with 55 °C (Q55). One unstressed (unexposed) isolate was maintained for each serotype as a positive control (C) yielding a total of 28 variants for further characterization (Figure 1).

### 2.2. Source of Salmonella Isolates and Preparation

The *Salmonella* isolates used were obtained from a pre-harvest poultry production study of broiler farms in our previous study [46], suggesting that they were not pre-exposed to sanitizers prior to use in this study. The isolates, maintained in tryptic soy broth (TSB) with glycerol at −80 °C, were streaked on tryptic soy agar (TSA) plates and incubated at 37 °C for 24 h. A single colony was then inoculated in TSB at 37 °C for 24 h and streaked on TSA plates to prepare working stocks that were stored at 4 °C and sub-cultured weekly. All the media used in this study were purchased from VWR International, LLC (Radnor, PA, USA) and manufactured by Hardy Diagnostics (Santa Maria, CA, USA), unless otherwise stated.

### 2.3. Stress Adaptation Study

The isolates were exposed to gradually increasing concentrations of PAA and QACs each day, starting at 40 ppm and 1 ppm, respectively until minimum inhibitory concentration (MIC) and minimum bactericidal concentration (MBC) were established. A preliminary micro-dilution MIC assay was conducted for each serotype prior to the stress adaptation study to determine the sub-inhibitory concentration (SIC) of the sanitizers. Therefore, these starting concentrations were the SICs of the antimicrobials and were sufficient to allow systematic, continuous, and daily conditioning without an immediate lethal effect on the isolates. Daily conditioning continued until the inhibitory and bactericidal thresholds were established for each serotype, and the duration (10–15 days) varied for each serotype. Once bacterial growth stopped, the stressed cells were collected from the pre-MBC plates and subjected to temperature-shock treatments at 4 °C for 60 min and 55 °C for 6 min. The variants generated along with their unstressed controls (Table 1) were then used to assess phenotypic expression, including homologous tolerance (MIC/MBC) to PAA and QACs, biofilm formation, anti-biofilm effects of PAA and QACs (biofilm recovery), and resistance profiling through antibiotic susceptibility testing.

### 2.4. Homologous Tolerance Profiling

To determine the tolerance of the variants (stressed and control) to PAA and QACs, overnight cultures containing approximately 10^8^ CFU/mL in TSB were standardized to a working inoculum of ~10^6^ CFU/mL. Each variant was tested against varying concentrations of PAA (40–150 ppm) and QACs (1–25 ppm). The concentration range, in 10 ppm and 1 ppm increments for PAA and QACs was streamlined to determine a close level of inhibitory threshold. The MIC was visually determined by turbidity after 24 h of incubation in 10 mL TSB containing PAA or QACs at 37 °C. The MIC was defined as the lowest concentrations of PAA and QACs required to inhibit the growth of the tested isolates. After MIC determination, the minimum bactericidal concentration (MBC) was assessed to evaluate the bactericidal effect of both PAA and QAC. Here, 100 µL from each tube showing no visible growth was plated onto XLD and TSA plates and incubated for 24 h at 37 °C. The MBC was the lowest concentration of PAA or QACs at which no bacterial growth was observed on the agar plates, indicating 99.9% killing effect on the organism.

### 2.5. Biofilm Formation Profiling

To assess biofilm formation on a plastic surface (96-well polystyrene plate, VWR), the variants were standardized to a working concentration of ~10^6^ CFU/mL and inoculated into 96-well polystyrene microtiter plates at 200 μL per well. Wells containing 200 μL of sterile TSB served as negative controls. The plates were incubated at 30 °C for 48 h to allow biofilm formation. Following incubation, the culture was removed, and each well was washed twice with 200 μL of sterile deionized water to remove planktonic cells. The plates were air-dried for 10 min and stained with 200 μL of 1% (*w*/*v*) crystal violet (Acros Organics, Thermo Fisher Scientific, Waltham, MA, USA) for 15 min. After staining, each well was washed three times with sterile deionized water to remove excess dye, then air-dried for 10 min before adding 200 μL of 95% ethanol (VWR) to dissolve the bound crystal violet for quantification [47,48]. Biofilm was quantified by measuring optical density (OD) at 600 nm with a spectrophotometer (Agilent Technologies, Inc., Santa Clara, CA, USA).

### 2.6. Biofilm Inactivation and Recovery Assay

To evaluate the recovery of each variant following exposure to the anti-biofilm activity of PAA and QACs, biofilms were formed and washed as previously described. Subsequently, 200 μL of either sterile phosphate-buffered saline (PBS) or the prepared solution of PAA at 110 ppm, 120 ppm, and 130 ppm or QACs at 11 ppm, 12 ppm, 14 ppm, 16 ppm, 18 ppm, and 20 ppm were added to the wells and incubated for 5 min at room temperature. These concentrations represent the MICs post-stress exposure. After treatment, the PBS and sanitizers were removed, and 200 μL of TSB containing sodium thiosulphate (a neutralizer) was added to assess post-exposure recovery of the variants. The plates were incubated at 30 °C for 24 h to assess biofilm recovery, and the OD was measured at 18 h using a spectrophotometer. Wells treated with PBS served as positive control in this assay.

### 2.7. Antibiotic Susceptibility Profiling

The variants were profiled for antibiotic resistance in accordance with NARMS (National Antimicrobial Resistance Monitoring System) guidelines [49]. Antibiotic susceptibility testing was performed using the broth microdilution method on the Sensititre™ Complete Automated AST System platform (Thermo Fisher Scientific Inc., Waltham, MA, USA) following the manufacturer’s instructions. Each antibiotic plate (CMV5AGNF) (Thermo Fisher Scientific Inc., Waltham, MA, USA) contained 13 antibiotics at concentrations representing the MIC breakpoints (Supplementary Table S1). The antibiotics tested were gentamicin (GEN), amoxicillin–clavulanic acid (AUG), cefoxitin (FOX), ceftriaxone (AXO), meropenem (MERO), ciprofloxacin (CIP), nalidixic acid (NAL), sulfisoxazole (FIS), trimethoprim/sulfamethoxazole (SXT), azithromycin (AZI), ampicillin (AMP), chloramphenicol (CHL), and tetracycline (TET). *Escherichia coli* ATCC 25922 was used as a reference strain for quality control. The bacterial suspension for each variant was prepared by inoculating 1 or 2 colonies into 5 mL demineralized water that was vortexed and adjusted to 0.5 McFarland standard using Sensititre^®^ nephelometer (Thermo Scientific^TM^) for inoculum standardization. A 10 µL of the suspension was then transferred to 11 mL of Mueller Hinton broth and vortexed thoroughly to make a final inoculum. Furthermore, 50 µL of the final inoculum was added to each well of the antibiotic plate using the automated Sensititre AIM^TM^ and the plate was incubated at 37 °C for 18 h. Plates were read using the Sensititre^®^ Vizion system, and AST results were interpreted based on NARMS-established MIC breakpoints and Clinical Laboratory Standards Institute (CLSI) guidelines (Table S1). Isolates with an increased MIC relative to the control were considered more tolerant (increased tolerance), whereas those with a reduced MIC were considered more susceptible (reduced tolerance) [49].

### 2.8. Statistical Analysis

The assays were performed in duplicate wells for each variant, and the experiments were replicated four times on separate days. The mean and standard deviation was calculated, and the data was analyzed using JMP Pro 18 (Cary, NC, USA) [50]. For comparisons within each *Salmonella* serotype (stressed variants against unstressed control) for each sanitizer, the data were analyzed using one-way analysis of variance (ANOVA), followed by Tukey’s honestly significant difference (HSD) test for pairwise comparisons. Comparisons and interactions between the serotypes and stress conditions (control, sanitizer stress alone, sanitizer with cold stress, and sanitizer with heat stress) were examined using a two-way ANOVA, and the means were separated using Tukey’s HSD. Normality assumptions were measured using residual Q–Q plots and homogeneity of variance of the *Salmonella* serotypes was assessed through visual inspection of residual plots. Statistical significance was defined as *p* ≤ 0.05.

## 3. Results

### 3.1. Tolerance of Salmonella Serotypes to Sanitizer Stress

Antibacterial (MIC) and bactericidal (MBC) activities of the sanitizers were determined following the daily exposure of *Salmonella* serotypes to PAA and QACs to assess tolerance. The MIC was determined by visual observation of growth inhibition, and the MBC was determined by plating aliquots from growth-negative tubes onto both XLD and TSA plates. After daily exposure, the MIC and MBC values for the PAA-stressed isolates ranged from 100 to 150 ppm, while the QAC-stressed isolates were 10 to 25 ppm (Table 2). After daily exposure to PAA stress, *S*. Kentucky had the lowest tolerance of 100 ppm (MIC and MBC), followed by *S*. Schwarzengrund and *S*. Infantis, which slightly increased to 110 ppm, but *S*. Infantis showed a 10 ppm difference between MIC (110 ppm) and MBC (120 ppm). Notably, *S*. Typhimurium showed the highest tolerance to PAA among the serotypes, with both MIC and MBC values reaching 150 ppm. QAC exposure, on the other hand varied between the serotypes. After QAC stress, *S*. Kentucky exhibited the highest tolerance in both MIC and MBC (25 ppm). At the same time, *S*. Infantis and *S*. Typhimurium showed elevated tolerance, with MICs and MBCs of 22 ppm and 20 ppm, respectively. Contrary to PAA stress, *S*. Schwarzengrund remained the most sensitive to QAC stress with both inhibition and elimination values remaining at 10 ppm.

### 3.2. Homologous Tolerance of Salmonella Variants

We evaluated the homologous tolerance of the stressed and control variants to PAA and QACs by exposing the variants to their respective sanitizer (i.e., all PAA variants to PAA and all QAC variants to QACs). All stressed variants of the four serotypes showed different tolerance compared to their unstressed controls (Table 3). There was a significant difference (*p* < 0.05) in the tolerance of the serotypes to stress conditions. Specifically, *S*. Kentucky demonstrated a significantly high tolerance, with MIC (120 ppm) and MBC (130 ppm) showing a 10–20 ppm difference compared to the control variant. Similarly, *S*. Infantis variants had a 17–20 ppm change in tolerance across the stressed variants for both MIC and MBC. Whereas *S*. Schwarzengrund only increased by 10 ppm compared to the control. Conversely, tolerance was stable at 120 ppm for the stressed variants of *S*. Typhimurium for both MIC (a 10 ppm difference) and MBC.

For QAC variants, exposure to QACs alone and in combination with temperature stress induced significant (*p* < 0.05) shifts in tolerance ranging from 2 to 8 ppm (Table 4). *S*. Kentucky presented the highest fold-increment in tolerance at 1.7-fold (MICs) and 1.4-fold (MBCs) compared to the control. Followed by *S*. Infantis and *S*. Typhimurium, which had 1.3 to 1.6-fold and 1.3 to 1.4-fold changes in MIC and MBC, respectively. Meanwhile, *S*. Schwarzengrund had the lowest response to QAC post-stress exposure, with no significant changes between the stressed and control variants.

### 3.3. Biofilm Formation of Salmonella Variants

**Figure 2:**
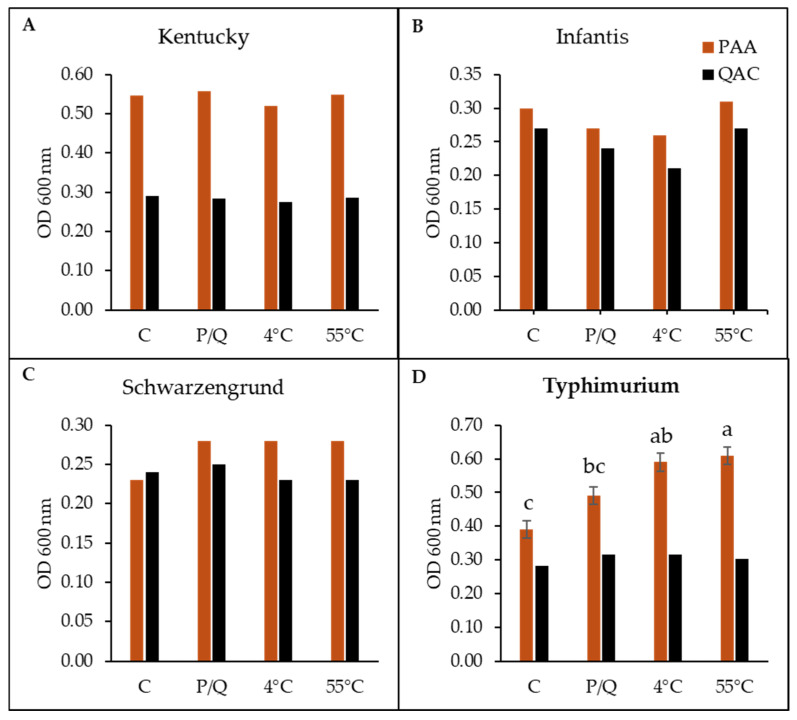
Biofilm formation of *Salmonella* variants after stress exposure for serotypes (**A**) Kentucky, (**B**) Infantis, (**C**) Schwarzengrund, and (**D**) Typhimurium on plastic surface. The variants include stressed with peracetic acid–PAA (P, colored bars) and quaternary ammonium compounds–QACs (Q, black bars), PAA/QAC-stressed plus temperature shock (P4/Q4 and P55/Q55), and the unstressed control (C). Data and error bars represent the average and standard error of the mean (SEM) of 4 replicates for each isolate. Superscripts indicate significant differences within serotypes for PAA (*p* < 0.0001, SEM = 0.01) and QACs (*p* = 0.0002, SEM = 0.01); and stress conditions for PAA (*p* = 0.002, SEM = 0.01) and QACs (*p* = 0.81, SEM = 0.01).

**Figure 3:**
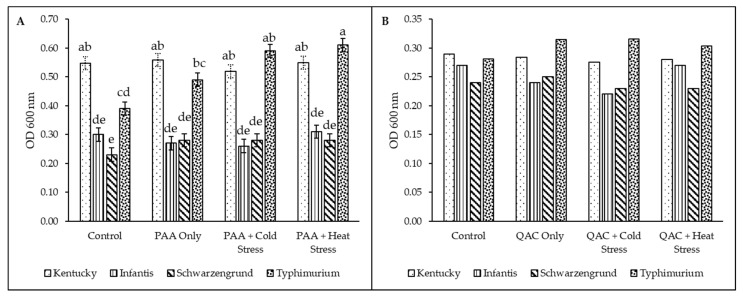
Summary of biofilm formation between *Salmonella* serotypes. (**A**) PAA variants and (**B**) QAC variants. Superscripts indicate significant differences for the interaction between serotypes and stress conditions for PAA (*p* < 0.0001, SEM = 0.02) and QACs (*p* = 0.80, SEM = 0.02).

To demonstrate any potential changes in biofilm-forming ability, all 28 variants were subjected to the crystal violet assay in a 96-well microtiter plate. Overall, there were significant differences (*p* < 0.05) among the serotypes, stress conditions, and the interaction between serotypes and stress conditions for PAA but not for QACs. Comparisons within each serotype revealed differences in biofilm formation among variants in response to PAA stress (*S*. Typhimurium alone) but not to QACs. There were no significant differences (*p* > 0.05) in biofilm formation between the stressed variant of *S*. Kentucky, *S*. Infantis, and *S*. Schwarzengrund and their controls for either sanitizer (Figure 2A–C). In fact, there was only a slight numerical difference in biofilm formation among the variants of *S*. Kentucky and *S*. Infantis for both PAA and QACs, with the stressed variant of *S*. Kentucky (P4) showing a slightly lower biofilm formation. In addition, there was a marginal reduction in biofilm formation for the PAA/QAC-stressed (P/Q) and PAA/QAC-stressed with 4 °C (P4/Q4) of *S*. Infantis. In contrast, the stressed variants of serotype Schwarzengrund showed slightly higher (PAA variants) and marginally lower (QAC variants) biofilm formation. However, the PAA-stressed *S*. Typhimurium variants produced significantly (*p* < 0.05, SEM = 0.03) more biofilm compared to the unexposed control (Figure 2D). Specifically, biofilm production was higher by roughly 1.5-fold in the combined stressed variants (i.e., P4 and P55) compared to the unstressed control and PAA-only stressed variants (P). Notably, this effect was not observed in the QAC variants (Figure 2D). Looking at differences in biofilm formation between the serotypes, the PAA variants significantly differed (*p* < 0.05, SEM = 0.02) (Figure 3A). In the unstressed control variants, *S.* Kentucky produced more biofilms, followed by *S.* Typhimurium and *S.* Infantis, while *S.* Schwarzengrund produced the least biofilm. This is slightly different from the PAA-only stressed variants in which serotypes Infantis and Schwarzengrund were similar, with lower biofilm formation than serotypes Kentucky and Typhimurium. The trend slightly shifted again for the double-stressed variants (PAA with either cold or heat stress), where *S*. Typhimurium numerically surpassed *S*. Kentucky in biofilm production (Figure 3A). These comparisons were not significant among the serotypes under QAC stress, although *S*. Kentucky and *S*. Typhimurium produced more biofilm across all groups from control to QAC-stressed (single and double) variants, with *S.* Typhimurium having the highest biofilm formation (Figure 3B).

### 3.4. Recovery of Salmonella Variants Post Antibiofilm Effect of Sanitizers

**Figure 4:**
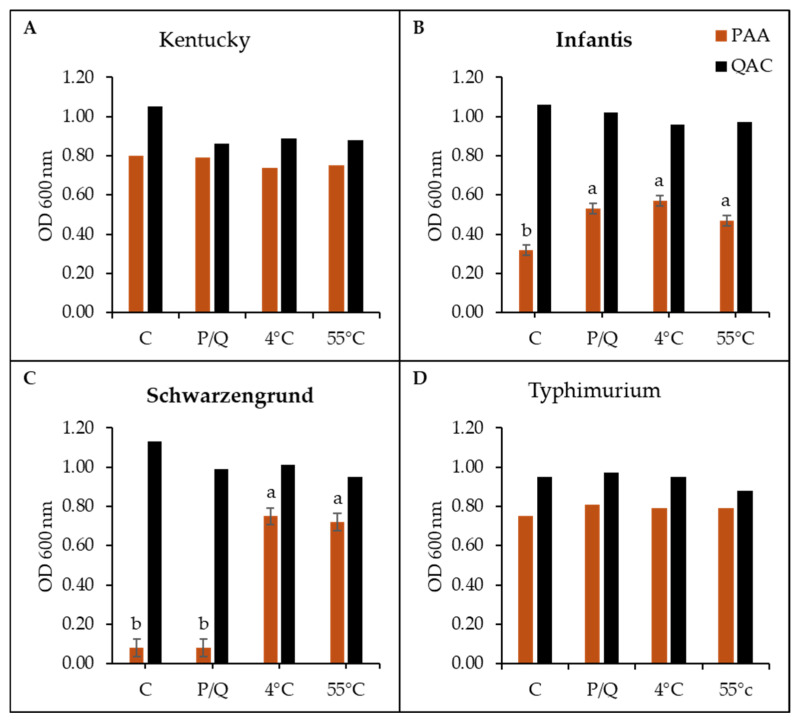
Antibiofilm effect of PAA and QACs on *Salmonella* variants of serotypes (**A**) Kentucky, (**B**) Infantis, (**C**) Schwarzengrund, and (**D**) Typhimurium on plastic surface. The variants include stressed with peracetic acid–PAA (P, colored bars) and quaternary ammonium compounds–QACs (Q, black bars), PAA/QAC-stressed plus temperature shock (P4/Q4 and P55/Q55, and the unstressed control (C). Data and error bars represent the average and standard error of the mean (SEM) of 4 replicates for each isolate. Superscripts indicate significant differences within serotypes for PAA (*p* < 0.0001, SEM = 0.02) and QACs (*p* = 0.0006, SEM = 0.02); and stress conditions for PAA (*p* < 0.0001, SEM = 0.02) and QACs (*p* = 0.0002, SEM = 0.02).

The efficacy of PAA and QAC stress exposure in enhancing biofilm regrowth of the stressed variants within and between *Salmonella* serotypes is presented in Figure 4A–D. PAA showed varying efficacy for biofilm inactivation across the stressed variants. While there was an overall significant difference (*p* < 0.05) between the serotypes, stress conditions, and their interactions for PAA; this was not consistent for QACs. There was no difference (*p* > 0.05) in the recovery of the variants of *S*. Kentucky and *S*. Typhimurium exposed to PAA (stressed versus control) (Figure 4A,D). In contrast, the PAA stressed variants of *S*. Infantis and *S*. Schwarzengrund showed significant biofilm regrowth post-PAA inactivation. For *S*. Infantis, all stressed variants showed a significant (*p* < 0.05, SEM = 0.03) increase in biofilm regrowth, with approximately doubled growth compared to the control variant (Figure 4B).

The double-stressed (P4 and P55) variants of *S*. Schwarzengrund exhibited an even greater response, with increased biofilm recovery, roughly a 9-fold increase (Figure 4C) compared to the PAA-stressed (P) and control variants that were similar in their regrowth. An intriguing finding was that QAC stress did not significantly enhance biofilm recovery/regrowth following QAC inactivation. Instead, all QAC-stressed variants across all serotypes exhibited slightly reduced growth compared to their unstressed counterparts (Figure 4A–D).

**Figure 5:**
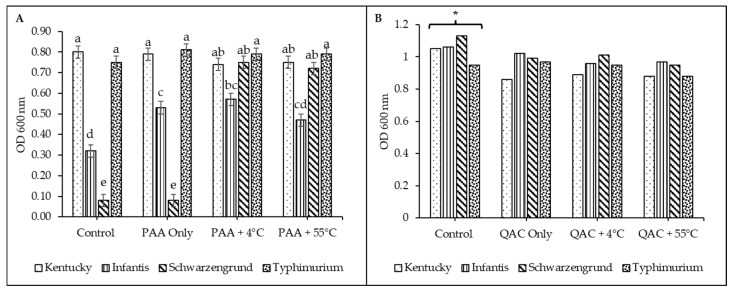
Summary of biofilm recovery between *Salmonella* serotypes. (**A**) PAA variants and (**B**) QAC variants. Asterisk (*) Indicate significant difference between serotypes. Superscripts indicate significant differences for the interaction between serotypes and stress conditions for PAA (*p* < 0.0001, SEM = 0.03) and QACs (*p* = 0.24, SEM = 0.04).

Following this, a comparison of biofilm recovery/regrowth between the serotypes is summarized in Figure 5A,B. Significantly, within the control and PAA-stressed groups, *S*. Kentucky and *S*. Typhimurium recovered more biofilm than *S*. Infantis and *S*. Schwarzengrund, which had the lowest recovery (Figure 5A). Whereas in the double-stressed groups, *S*. Infantis consistently had the lowest biofilm recovery. For QAC variants, biofilm formation of the serotype was similar across all groups (stressed and controls), but *S*. Kentucky showed slightly less (*p* > 0.05) biofilm regrowth in the stressed groups (Figure 5B).

### 3.5. Changes in Resistance Profiles of Salmonella Variants

**Figure 6:**
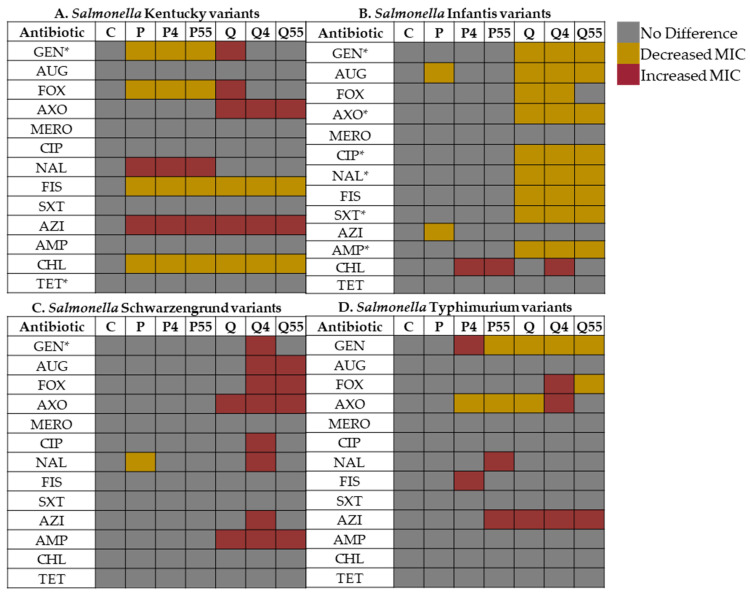
Antibiotic susceptibility of *Salmonella* variants of serotypes (**A**) Kentucky, (**B**) Infantis, (**C**) Schwarzengrund, and (**D**) Typhimurium. Antibiotics tested include GEN: Gentamicin, AUG: Amoxicillin–Clavulanic Acid, FOX: Cefoxitin, AXO: Ceftriaxone, MERO: Meropenem, CIP: Ciprofloxacin, NAL: Nalidixic Acid, FIS: Sulfisoxazole, SXT: Trimethoprim–Sulfamethoxazole, AZI: Azithromycin, AMP: Ampicillin, CHL: Chloramphenicol, and TET: Tetracycline. Interpretation was based on NARMS guidelines. An asterisk (*) next to the antibiotics means the control (C) was already resistant. The gray box indicates the MIC is the same as the control (i.e., no change in MIC), the yellow box indicates the MIC decreased compared to the control (i.e., reduced tolerance), and the brown box indicates the MIC increased compared to the control (i.e., increased tolerance).

All variants were exposed to 13 antibiotics, and interestingly, the QAC-stressed variants showed more phenotypic changes than their PAA-stressed counterparts across the 24 stressed variants (Figure 6A–D). The antibiotic concentration range with MIC interpretive standards according to NARMS guidelines is provided in Supplementary Table S1, and the MICs for the *Salmonella* variants interpretations are in Table S2. For *S*. Kentucky, the control variant showed natural resistance to gentamicin and tetracycline (Figure 6A). All the stressed variants became less tolerant (lower MICs) to gentamicin and cefoxitin (PAA variants only), sulfisoxazole, and chloramphenicol. At the same time, there was increased tolerance to other antibiotics, including ceftriaxone by a 2-fold increment (QAC variants), nalidixic acid (PAA variants), and azithromycin (all stressed variants). The MICs for sulfisoxazole were reduced by half compared to the control, while chloramphenicol declined by half under PAA stress and a quarter under QAC stress, leading to a shift from intermediate to susceptible category (Tables S1 and S2). All stressed variants showed increased tolerance to azithromycin but remained within the susceptible range of the NARMS breakpoint (Table S2).

*S*. Infantis showed greater natural resistance to the antibiotics tested, including gentamicin, ceftriaxone, ciprofloxacin, nalidixic acid, trimethoprim–sulfamethoxazole, and ampicillin (Figure 6B). Here, the PAA variants showed little to no change in tolerance (chloramphenicol only for P4, P55, and Q4) compared to their QAC counterparts that had increased susceptibility (lower MIC) to nine of the 13 antibiotics tested. These include clinically important antibiotics such as ceftriaxone and cefoxitin, with no change in azithromycin. In the QAC-stressed variants, MICs for gentamicin and ciprofloxacin were reduced from intermediate to susceptible (Table S2), and a similar trend was observed in ceftriaxone, nalidixic acid, trimethoprim–sulfamethoxazole, and ampicillin, which changed from resistance to susceptible (Figure 6B, Table S2).

In contrast, the resistance of *S*. Schwarzengrund showed a mostly upward trend as natural resistance was only to gentamicin (Figure 6C). Increased tolerance for many of the antibiotics was observed in the QAC variants alone, especially the QACs plus cold-stressed variant (Q4) that showed the most dramatic change to eight antibiotics, especially nalidixic acid, which was 8-fold higher than the control variant, resulting in a significant phenotypic alteration from susceptible to resistant (Table S2). At the same time, the MIC for azithromycin was augmented by 4-fold but did not exceed NARMS susceptible threshold (Table S1).

As shown in Figure 6D, *S*. Typhimurium was susceptible to all antibiotics tested and generally had the lowest phenotypic changes. There was a sporadic shift among some of the antibiotics, including reduced MICs for the stressed variants (mostly QACs) to gentamicin (P55 and all QACs), cefoxitin (Q55 only), and ceftriaxone (P4, P55, and Q). There was increased tolerance for gentamicin and sulfisoxazole (P4), nalidixic acid and azithromycin (P55), cephalosporins (cefoxitin and ceftriaxone, Q4), and azithromycin (all QAC variants). The elevated MIC for azithromycin in the QAC variants was remarkable, doubling that of the control, but did not surpass the NARMS susceptibility breakpoint (Tables S1 and S2).

## 4. Discussion

This study evaluated phenotypic alterations to *Salmonella enterica* as an adaptive response to environmental stress. The results suggest that exposure to environmental stressors such as sanitizers and temperature can significantly influence the behavior/response of *Salmonella enterica*. We showed that continuous exposure to sublethal doses of PAA or QACs increased the inhibitory and bactericidal thresholds of the strains tested in this study compared with their unstressed controls. When these stressed variants were further challenged with cold or heat shock (stress), the tolerance pattern mostly remained the same, suggesting that combined stress conditions may potentially act synergistically to influence bacterial tolerance. Several studies have reported that continuous or daily exposure to sub-lethal concentrations of sanitizers can lead to bacterial tolerance as an adaptive response [51,52,53,54]. While limited data exists on bacterial adaptive response to PAA, all stressed variants of the four serotypes tested in our study increased their tolerance to PAA, the most common peroxide-based sanitizer used in post-harvest poultry processing [33]. The variants also showed changes in tolerance to QACs, a commonly used sanitation agent. These results are consistent with those of others who reported changes in MIC when *Salmonella* strains were exposed to sanitizer stress [39,41,43,44]. PAA stress adaptation, though not extensively studied, may be attributed to its oxidative nature, which causes cellular injury (stress) by accumulating reactive oxygen species (ROS) that damage proteins, lipids, and DNA [55]. Bacteria can temporarily tolerate exposure to ROS during oxidative stress, thereby causing injury rather than death by activating the oxidative stress response genes, including SoxRS, OxyR and PerR regulons [56]. For QACs like benzalkonium chloride, stable tolerance is often mediated by an efflux pump, induced by the transcriptional activator RamA, which activates the AcrAB-TolC multidrug efflux pump [57,58]. This efflux pump actively expels harmful substances from the cell, thereby conferring resistance [58]. Cetylpyridinium chloride is another QAC that acts on bacteria by disrupting cell membrane, displacing stabilizing cations, and inserting its hydrophobic tail into the lipid bilayer, thereby causing membrane destabilization, functional impairment, and ultimately cell lysis [59,60]. These sanitizers not only induce tolerance but also promote biofilm formation, contributing to the environmental persistence of *Salmonella* [54].

There were major differences in biofilm formation among all serotypes tested in this study, with *S.* Kentucky (control variant) generally producing more biofilms (1.5 to 2-fold) than the other serotypes, which could explain the lack of changes in biofilm formation among the stressed variants. For instance, if the strain was already a moderate to strong biofilm producer, then stress exposure would not further enhance its biofilm formation. While this argument could be held for *S.* Kentucky, it does not for *S.* Infantis and *S*. Schwarzengrund that produced little to no biofilm (control variants), and the biofilm formation did not change after stress. Notably, *S.* Typhimurium (control variant) was a weak biofilm producer, and stress exposure, especially double stress shifted its biofilm formation in the PAA-stressed variants. Our findings are consistent with those of Dhakal et al. [61], who reported that stressed *S.* Typhimurium (ATCC 14028) exhibited higher biofilm formation compared to the non-stressed control, and those of Obe et al. [48], who showed significant differences in many *Salmonella* serotypes and even strains of the same serotype. While Obe et al. [48] found strong biofilm formation in *S*. Schwarzengrund strains tested under multiple conditions, we did not observe such affinity in our study, as the strain used here was a weak biofilm producer. Furthermore, Castelijn et al. [62] reported variability in biofilm-forming capacity among isolates within serotypes Typhimurium and Infantis, with production ranging from weak to strong, unlike in serotypes Brandenburg and Derby, where over 80% of the isolates were strong and weak producers, respectively. This diversity suggests that adaptive responses and persistence of *Salmonella enterica* can be strain-dependent, as strong biofilm-forming strains are better equipped to withstand higher sanitizer pressure, contributing to their persistence in processing environments [29]. A significant difference between some of these previous studies and ours lies in the source of the strains used. The strains used in this study are presumed not to have been exposed to any sanitizer, although they were obtained from a poultry farm environment (pre-harvest), which could explain the weak to moderate biofilm formation observed in the control variants. In contrast, other studies used strains obtained from processing plants, humans, and foodborne outbreaks that have certainly been exposed to sanitizers and disinfectants or have survived rigorous environmental conditions.

We further assessed the effectiveness of these sanitizers in inactivating biofilms formed by the tested variants as well as their survival after treatment. Overall, PAA reduced bacterial recovery across all tested serotypes compared to QACs. Like biofilm formation, *S.* Kentucky recovered more abundantly than other serotypes, perhaps due to its moderate biofilm formation, suggesting that PAA was less effective against the biofilm variants–both control and stressed. The variants of *S.* Typhimurium also showed reduced PAA efficacy, as their regrowth was like that of *S*. Kentucky and higher than that observed during biofilm formation. Contrarily, the regrowth of stressed variants of *S.* Infantis showed a slight increase (~2-fold) compared to the control variant and during biofilm formation, while those of *S.* Schwarzengrund, particularly the double-stressed variants, recovered abundantly (8-fold increment). *S.* Schwarzengrund may have a stronger affinity for extreme adaptive responses when pre-exposed to multiple environmental stresses, as seen in a previous study where *S.* Schwarzengrund strains produced more biofilm [48]. On the other hand, QACs demonstrated very low antibiofilm activity against all serotypes. Specifically, biofilm formation of the serotypes ranged from 0.2 to 0.3 (OD_600_ nm), while biofilm regrowth post-QAC exposure increased to about 3-fold across the variants. Other studies have reinforced the idea of PAA’s vigorous antimicrobial activity compared to other agents such as QACs and chlorine [63,64]. The superior anti-biofilm effect of PAA could be attributed to slow reaction with organic matter and its ability to penetrate biofilm matrix components, allowing greater stability and prolonged exposure time [65,66]. Multiple adaptive mechanisms can explain the lower anti-biofilm effect of QACs. Some *Salmonella* strains harbor QAC-tolerance genes (e.g., the *qac* family) that encode efflux pumps that actively remove QAC molecules from the cell. Furthermore, changes in membrane structure and upregulation of efflux pump activity further enhance bacterial tolerance to QACs, contributing to biofilm survival, persistence, and regrowth [67].

The tolerance of the variants was further assessed by their response to antibiotic treatment at varying concentrations. Differences in tolerance were considerably evident between the serotypes and among variants within each serotype. We observed that *S*. Infantis had the highest resistance profile to gentamicin, ceftriaxone, nalidixic acid, trimethoprim–sulfamethoxazole, and ampicillin. This finding is consistent with previous research indicating that *S*. Infantis isolates exhibited high resistance to ceftriaxone, nalidixic acid, trimethoprim–sulfamethoxazole, and ampicillin [68]. This high resistance can be explained by the emergence of strains harboring a unique pESI megaplasmid, an IncFIB-type plasmid that originated through multiple recombination events [23,69]. This megaplasmid harbors genes encoding fimbriae, siderophore-iron transport system, heavy-metal resistance, and antibiotic resistance, particularly blaCTX-M-65, which is present in approximately half of pESI plasmids [70]. The *S*. Infantis isolate used in our study may harbor this megaplasmid. However, it was obtained from pre-harvest poultry production and had no opportunity to express this virulent potential. Regardless, it is essential to note that exposure to environmental stress can enhance (upregulate or downregulate) this natural tolerance. Our study revealed greater stress-induced shifts in antibiotic resistance patterns, described as increases in MICs following PAA stress and in combination with temperatures, mainly in *S.* Kentucky (nalidixic acid and azithromycin) and slightly in *S.* Typhimurium (gentamicin, nalidixic acid, sulfisoxazole, and azithromycin). Considering these two serotypes, PAA stress (alone and combined) led to a greater decrease in tolerance (i.e., reduced MICs and increased susceptibility), particularly in *S.* Kentucky, across many of the antibiotics tested. At the same time, PAA stress did not affect the antibiotic resistance of *S.* Infantis and *S.* Schwarzengrund. To date, too little attention has been paid to the relationship between PAA and antibiotic resistance. Previous studies have demonstrated that repeated exposure to PAA enhanced resistance to tiamulin in *Streptococcus suis* [71]. The changes in antibiotic resistance patterns might be explained by the presence of plasmid-borne antibiotic resistance genes or DNA that remain intact and functional after the disinfection process, because they are protected by cellular structures like the cell membrane and cytoplasm [72], allowing transforming activity and antibiotic resistance [73]. This could further allow their persistence as residual plasmid DNA or genes and possible dissemination of resistance genes in the environment via horizontal gene transfer.

On the contrary, QAC stress notably resulted in reduced tolerance in *S.* Infantis compared to the other serotypes, while increasing the tolerance of *S.* Kentucky and, to a greater extent *S.* Schwarzengrund. This is an interesting finding that warrants further evaluation of genotypic differences and strain-to-strain variation within each serotype. For all other phenotypic assays evaluated, QACs did not result in a notable change in the tested strains, as observed for antibiotic resistance. The rise in antibiotic resistance to QACs can be explained by the presence of mobile genetic elements (plasmids, transposons, and integrons) carrying multiple genes encoding resistance to QACs or antibiotics, further supporting the possibility of co-resistance mechanisms [67,74]. Recent studies reveal that widespread use of disinfectants can promote the horizontal transfer of antibiotic-resistant genes, leading to the dissemination of antibiotic resistance among bacteria [71].

Furthermore, bacteria can acquire resistance to disinfectants and antibiotics through co-selection, including co-resistance, cross-resistance, and co-regulatory resistance [75]. The phenomenon of co-resistance occurs when antibiotic and disinfectant resistance genes are co-located on a single mobile genetic element, enabling simultaneous transfer [71]. In addition to sanitizer stress, previous studies have shown that exposure to cold stress alters the membrane structure of *S*. Typhimurium, leading to variations in antibiotic resistance levels [76]. Likewise, Qiao et al. [77] reported that cold stress could induce target-site mutations (e.g., in the GrlA subunit), thereby diminishing antibiotic binding efficiency and ultimately increasing resistance. Following cold stress, heat stress can also similarly induce antibiotic resistance. Furthermore, changes in MIC or antibiotic resistance profile may be due to alterations in membrane fluidity, expression of heat shock proteins, and modification of receptor-binding sites that influence antibiotic transport under heat stress [78].

A caveat to our study is that we did not pre-evaluate the genetic profiles of the isolates tested (control variants) to assess the extent to which these stress conditions could alter the strains, particularly as seen in *S.* Infantis, which was naturally resistant to many of the antibiotics. Interestingly, this allowed us to understand the impact of pre-exposure of *Salmonella enterica* strains to processing conditions on adaptation or antimicrobial resistance outcomes. Many studies have used bacterial isolates obtained from processing/retail environments or from foodborne outbreaks, which have been pre-exposed to environmental stress and explains their responses to further stress under controlled conditions. Furthermore, the phenotypic responses evaluated in our study were under single antimicrobial stress (PAA or QACs), which do not fully capture the complexity and dynamic nature of the environmental stress encountered by *Salmonella* and other foodborne pathogens in processing environments. However, bacteria are routinely subjected to multiple antimicrobial interventions simultaneously in commercial food processing plants, potentially eliciting distinct adaptive responses. Future studies are needed to examine the combined effects of multiple antimicrobial stresses across *Salmonella* serotypes.

## 5. Conclusions

Our study found that environmental stress can increase *Salmonella* survival and persistence by enhancing sanitizer tolerance, improving biofilm formation, and promoting recovery post-sanitation. Moreover, these responses could influence the resistance of persistent strains to clinically relevant antibiotics through cross-resistance mechanisms, posing significant challenges to the control of *Salmonella* in the processing environments and for public health. The sanitizers used in this study, especially PAA, are routinely used in poultry processing environments, and, to the best of our knowledge, PAA is being evaluated for the first time to induce adaptive responses and tolerance across multiple *Salmonella* serotypes, suggesting a need for appropriate use of these chemicals. Further studies are warranted, however, to better understand the genetic diversity across the variants studied here, elucidate mechanisms underlying adaptive responses and antibiotic resistance, and identify additional procedures that could be modified in processing environments to control bacterial adaptation.

## Figures and Tables

**Table 1:** Variants of *Salmonella* serotypes tested for phenotypic expression.

Serotypes	Variants
Kentucky	Control (C) *
Infantis	PAA (P) or QAC (Q)-stressed
Schwarzengrund	PAA/QAC stress + cold shock (P4) or (Q4)
Typhimurium	PAA/QAC stress + heat shock (P55) or (Q55)
	6 variants × 4 serotypes = 24 stressed variants With 4 controls = 28 variants

* Control (C) is unstressed/unexposed control to understand the impact of antimicrobial exposure/stress.

**Table 2:** Tolerance of *Salmonella* serotypes to daily conditioning with PAA and QACs.

	PAA Stress (ppm)		QAC Stress (ppm)	
Serotypes	MIC	MBC	MIC	MBC
Kentucky	100	100	**25**	**25**
Infantis	110	120	22	22
Schwarzengrund	110	110	10	10
Typhimurium	**150**	**150**	20	20

The minimum inhibitory concentrations (MICs) and bactericidal concentrations (MBCs) of the sanitizers, PAA (peracetic acid) and QACs (quaternary ammonium compounds) in parts per million (ppm) were established using broth micro-dilution assay prior to systematic stress exposure experiments through daily conditioning. The bold data represent the highest MIC/MBC across the serotypes after daily conditioning.

**Table 3:** Tolerance of post-stressed variants of *Salmonella* serotypes to PAA (ppm).

	Unstressed		PAA Stressed (P)		PAA + Cold Stress (P4)		PAA + Heat Stress (P55)	
Serotypes	MIC	MBC	MIC	MBC	MIC	MBC	MIC	MBC
Kentucky	100 ^C^	110 ^C^	120 ^A^	130 ^A^	120 ^A^	130 ^A^	120 ^A^	130 ^A^
Infantis	92.5 ^D^	105 ^C^	110 ^B^	125 ^AB^	110 ^B^	125 ^AB^	110 ^B^	125 ^AB^
Schwarzengrund	100 ^C^	120 ^B^	110 ^B^	130 ^A^	110 ^B^	130 ^A^	110 ^B^	130 ^A^
Typhimurium	110 ^B^	120 ^B^	120 ^A^	120 ^B^	120 ^A^	120 ^B^	120 ^A^	120 ^B^

The minimum inhibitory (MICs) and bactericidal (MBCs) concentrations of the PAA (peracetic acid) in parts per million (ppm) after post-stress exposure experiment. Superscripts (A to D) are independent for each of MICs and MBCs and indicate significant interactions between the serotypes and stress conditions (MICs: *p* < 0.0001, SEM = 0.62; MBCs: *p* < 0.0001, SEM = 1.44). SEM (standard error of the mean).

**Table 4:** Tolerance of post-stressed variants of *Salmonella* serotypes to QACs (ppm).

	Unstressed		QAC Stressed (Q)		QAC + Cold Stress (Q4)		QAC + Heat Stress (Q55)	
Serotypes	MIC	MBC	MIC	MBC	MIC	MBC	MIC	MBC
Kentucky	12 ^CD^	14 ^D^	20 ^A^	20 ^B^	20 ^A^	20 ^B^	20 ^A^	20 ^B^
Infantis	12.5 ^C^	17.5 ^C^	20 ^A^	25 ^A^	20 ^A^	25 ^A^	20 ^A^	25 ^A^
Schwarzengrund	9 ^E^	10.3 ^E^	10.8 ^CDE^	11.8 ^E^	10.5 ^DE^	11.8 ^E^	10.3 ^DE^	11.5 ^E^
Typhimurium	12 ^CD^	14 ^D^	16 ^B^	18 ^C^	16 ^B^	18 ^C^	16 ^B^	18 ^C^

The minimum inhibitory (MIC) and bactericidal (MBC) concentrations of the PAA (peracetic acid) in parts per million (ppm) after post-stress exposure experiment. Superscripts (A to E) are independent for MIC and MBC and indicate significant interactions between the serotypes and stress conditions. (MIC: *p* < 0.0001, SEM = 0.38; MBC: *p* < 0.0001, SEM = 0.38). SEM (standard error of the mean).

## Data Availability

Data available on request from the corresponding author.

## Supplementary Materials

The following supporting information can be downloaded at: https://www.mdpi.com/article/10.3390/microorganisms14040748/s1, Table S1: Antibiotics concentration range and MIC interpretive standard (μg/mL) according to NARMS guideline; Table S2: Interpretation of the MIC (μg/mL) of the antibiotic susceptibility test.

## Abbreviations

The following abbreviations are used in this manuscript:

AMP	Ampicillin
AMR	Antimicrobial Resistance
ANOVA	Analysis of Variance
AUG	Amoxicillin–Clavulanic Acid
AXO	Ceftriaxone
AZI	Azithromycin
CDC	Centers for Disease Control and Prevention
CFU	Colony Forming Unit
CHL	Chloramphenicol
CIP	Ciprofloxacin
ESBL	Extended-Spectrum Beta-lactamase
FIS	Sulfisoxazole
FOX	Cefoxitin
FSIS	Food Safety Inspection and Services
GEN	Gentamicin
MBC	Minimum Bactericidal Concentration
MERO	Meropenem
MIC	Minimum Inhibitory Concentration
NAL	Nalidixic Acid
NARMS	National Antimicrobial Resistance Monitoring System
PAA	Peracetic Acid
PBS	Phosphate-Buffered Saline
QACs	Quaternary Ammonium Compounds
SXT	Trimethoprim–Sulfamethoxazole
TET	Tetracycline
TSA	Tryptic Soy Agar
TSB	Tryptic Soy Broth
USDA	United States Department of Agriculture
XLD	Xylose Lysine Deoxycholate
